# The Effect of Training-Induced Visual Imageability on Electrophysiological Correlates of Novel Word Processing

**DOI:** 10.3390/biomedicines6030075

**Published:** 2018-07-01

**Authors:** Laura Bechtold, Marta Ghio, Christian Bellebaum

**Affiliations:** Institute for Experimental Psychology, Heinrich Heine University Düsseldorf, 40225 Düsseldorf, Germany; Marta.Ghio@hhu.de (M.G.); Christian.Bellebaum@hhu.de (C.B.)

**Keywords:** N400, N700, concreteness, imageability, novel words, learning

## Abstract

The concreteness effect (CE) describes a processing advantage for concrete over abstract words. Electrophysiologically, the CE manifests in higher N400 and N700 amplitudes for concrete words. The contribution of the stimulus-inherent imageability to the electrophysiological correlates of the CE is not yet fully unraveled. This EEG study focused on the role of imageability irrespective of concreteness by examining the effects of training-induced visual imageability on the processing of novel words. In two training sessions, 21 healthy participants learned to associate novel words with pictures of novel objects as well as electron-microscopical structures and were additionally familiarized with novel words without any picture association. During a post-training EEG session, participants categorized trained novel words with or without picture association, together with real concrete and abstract words. Novel words associated with novel object pictures during the training elicited a higher N700 than familiarized novel words without picture-association. Crucially, this training-induced N700 effect resembled the CE found for real words. However, a CE on the N400 was found for real words, but no effect of imageability in novel words. The results suggest that the N400 CE for real words depends on the integration of multiple semantic features, while mere visual imageability might contribute to the CE in the N700 time window.

## 1. Introduction

Language processing requires an association of a word’s form with its referent’s conceptual representation in semantic memory. Conceptual representations combine information taken from learning experience with the word and/or its referent and provide this information in the course of conceptual processing [1,2]. Depending on experiential differences concerning the words’ referents, words are often classified as either concrete or abstract (for a review see [3]). Concrete words’ referents (e.g., hammer) are perceivable with the external bodily senses. Abstract words refer to states or entities (e.g., harmony), which are not directly perceivable via external bodily senses, but rather arise from lexical information [4] or internal bodily senses (e.g., mental or emotional experience) [3,5,6,7].

The concreteness effect (CE) describes a processing advantage for concrete over abstract words in memory, comprehension and production tasks (for reviews see [3,8,9]). The dual-coding theory [4] explains the CE in terms of richer conceptual representations of concrete words, based on sensory as well as lexical information, while representations of abstract words rely on lexical information only. The context availability model [8] attributes the CE to an easier retrieval of a greater amount of conceptual information for concrete than abstract words. By now, novel approaches integrate dual-coding and context availability (e.g., the extended dual-coding theory) [10,11], as they seem to highlight the two distinct but compatible semantic processes of concept representation and retrieval, which rely on different interacting neural correlates [3,12].

One component considered crucially relevant for processing differences between concrete and abstract words is their imageability. Dual-coding as well as context availability accounts assign concrete words a higher imageability in terms of conceptually integrated visual sensory information [9] and accessibility of mental images [13], respectively. Language processing advantages driven by imageability have been shown in children, who initially acquire [14,15,16] and subsequently learn to read [17] highly imageable words earlier and better. Moreover, concrete and/or highly imaginable word processing is often less severely impaired by clinical disorders like semantic dementia [18,19], dyslexia [20,21,22,23,24,25] and Alzheimer’s disease [26]. Kellogg, et al. [27] showed that a concurrent visual working memory task impaired the performance of healthy young adults in a definition production task for concrete but not abstract words, further supporting the role of imageability in the CE. Therefore, it is not surprising that concreteness and imageability ratings are highly correlated [28] and the two terms are often used interchangeably [3,29].

In electroencephalography (EEG) studies, the CE becomes manifest in higher amplitudes of the N400 and N700 event-related potential (ERP) components for concrete in comparison to abstract words [30]. The N400 has been interpreted to reflect the strength of the activation of the semantic network and integration of semantic information (for extensive reviews see [31,32]). The frontally pronounced N700 has been linked to mental imagery processes [10,30,33,34,35]. Findings of Barber, et al. [36] question the role of imageability for the electrophysiological CE. They matched concrete and abstract words for imageability and found a reversed behavioral CE but still higher N400 amplitudes for concrete than abstract words. Therefore, the effects of imageability and concreteness on word processing seem to be at least partially dissociable.

One recent line of research made important contributions for disentangling the effects of imageability and concreteness on N400 and N700 amplitudes. Concrete and abstract words (as stand-alone stimuli, see [35]; or in a sentential context, see [34,37]) were processed in an image generation as well as in a lexical and/or surface-level processing task in order to manipulate stimulus- and task-driven imagery processes, respectively. Altogether, these studies suggest that word concreteness and imageability are distinct semantic features, which are integrated in the processing stage reflected by the N400. At the later processing stage reflected by the N700, mental images of words might be generated, but only when the task as well as the stimuli afford it (for a detailed discussion and information on methodological differences see [35]). Gullick, Mitra and Coch [35] interpret their findings based on the extended dual-coding theory [10,11], and suggest that concreteness is not merely relying on sensorimotor information but includes lexically mediated information as well, while imageability is derived from (in their case visual) sensory information alone. In order to test this assumption, one could investigate the contribution of word imageability and concreteness to N400 and N700 modulations separately by employing stimuli with just one or the other semantic feature.

This study aimed at investigating the extent to which visual imageability untainted by concreteness modulates the N400 and N700 by using formerly meaningless, novel words that were either associated with visual stimuli during a training phase or not. In particular, in a two-day training, subjects learned to associate novel words with pictures of novel, unknown objects (OPic; see [38]) or of electron-microscopical structures (SPic). The two types of pictorial stimuli were chosen because of the different types of visual information they provide and were thus expected to lead to differences in imageability between the associated word stimuli. More specifically, the OPic were expected to lead to higher imageability than the SPic as we chose them to more distinctively depict one coherent entity. As a control condition, participants learned novel words that were not associated with any visual stimulus (NoPic). We thereby manipulated the novel words’ imageability, without introducing any additional (lexical or sensory) information possibly contributing to the CE [3,39,40,41]. In a post-training EEG session, we examined the processing of the novel words while participants performed a concreteness-judgment task.

The results for the N400 could help to elucidate the role of imageability for the CE. If imageability itself contributes to the N400 CE, training-induced higher visual imageability should lead to higher N400 amplitudes. If, however, the N400 CE depends on an integrative interaction of sensorimotor and lexically coded features underlying word concreteness [32,35,42], no effect should be seen at this processing stage. For the N700, we expected to see larger amplitudes for higher imageability, as the task used in our study was designed to afford imagery processes [35]. Finally, as the effect of visual imageability on word processing presumably contributes to the electrophysiological CE, we presented concrete and abstract real words intermixed with the novel words in our study. The aim was to elicit a classical CE in the N400 and N700 time windows within the same experimental paradigm and qualitatively compare the CE in real words with the effects of imageability on novel words.

## 2. Materials and Method

### 2.1. Participants

Twenty-four healthy German native speakers (age from 19 to 34 years) took part in the study. Three participants were excluded from the analyses due to a poor learning performance and thus too few trials for the EEG analyses (<20 for at least one experimental condition). The remaining 21 participants (10 women; mean age = 24.8 years, *SD* = 4.1 years) had normal or corrected to normal vision and were right handed as indicated by the Edinburgh Handedness Inventory [43] (scores between 0.55 and 1, *M* = 0.88, *SD* = 0.14). All participants gave their written informed consent. After participation, they received course credit or monetary compensation. This study was in line with the declaration of Helsinki and was approved by the ethics committee of the Faculty of Mathematics and Natural Sciences.

### 2.2. Material

#### 2.2.1. Visual Stimuli

The visual stimuli were 8-bit color JPG images of 15 unfamiliar objects (OPics) and 15 electron-microscopical pictures of structures (SPics). The objects were built of a construction toy (K’NEX^TM^) and had already been used in previous training studies [38,44,45,46,47]. For each object, photographs from four isometric perspectives were available. The electron-microscopical pictures were acquired via google image search and consisted of different living and non-living structures (e.g., legionella, rocks, asbestos, skin). They were each cut into four partially overlapping segments and a slight vignette, extracted from the object picture backgrounds, was added. Electron-microscopical images were originally monochrome. The color information of the OPics was extracted, smoothed and transferred onto the SPics via the GNU Image Manipulation Program (GIMP, version 2). The mean brightness (measured with the pictures’ histograms, from 0 = white to 255 = black with GIMP) of the 60 OPics (*M* = 176.77, *SD* = 8.49) and 60 SPics (*M* = 176.77, *SD* = 8.46) was carefully matched, *t*(118) = 0.001, *p* = 0.999.

#### 2.2.2. Verbal Stimuli

The 60 word-like pseudo-words used as novel words in this study were created by changing two to three letters in real German words, following phonological rules (e.g., Himmar, Neribon). This pool of stimuli was divided into four subsets, each including 15 words. Each subset was assigned to one of the experimental conditions, namely OPic, SPic, NoPic (familiarized in the training but without associated pictures, served as a lexical baseline condition) and New (only used as filler stimuli for the EEG task, see below). The novel words in these four subsets were matched for the number of letters (*M*_OPic_ = 7.67, *SD*_OPic_ = 0.90; *M*_SPic_ = 7.67, *SD*_SPic_ = 0.90; *M*_NoPic_ = 7.73, *SD*_NoPic_ = 0.88; *M*_New_ = 7.87, *SD*_New_ = 0.92; Kruskal-Wallis-test for independent samples, *H*(3) = 0.250, *p* = 0.969) and syllables (*M*_OPic_ = 2.53, *SD*_OPic_ = 0.52; *M*_SPic_ = 2.53, *SD*_SPic_ = 0.52; *M*_NoPic_ = 2.53, *SD*_NoPic_ = 0.52; *M*_New_ = 2.47, *SD*_New_ = 0.52; Kruskal-Wallis-test for independent samples, *H*(3) = 0.197, *p* = 0.978). The 60 real words additionally used in the EEG concreteness-judgment task consisted of 30 concrete and 30 abstract words. They were also matched for the number of letters (*M*_concrete_ = 7.07, *SD*_concrete_ = 1.02; *M*_abstract_ = 7.10, *SD*_abstract_ = 1.79; Kruskal-Wallis-test for independent samples, *H*(1) = 0.052, *p* = 0.820) and syllables (*M*_concrete_ = 2.40, *SD*_concrete_ = 0.50; *M*_abstract_ = 2.47, *SD*_abstract_ = 0.51; Kruskal-Wallis-test for independent samples, *H*(1) = 0.267, *p* = 0.605). Concrete and abstract real words were additionally matched for their lexical frequency as assessed via a word database of the university of Leipzig, (http://wortschatz.uni-leipzig.de, 20 March 2015; *M*_concrete_ = 1726.68, *SD*_concrete_ = 1698.92; *M*_abstract_ = 1746.57, *SD*_abstract_ = 2124.28; Kruskal-Wallis-test for independent samples, *H*(1) = 0.514, *p* = 0.473). All real words were rated regarding eight different psycholinguistic variables (Concreteness, Imageability, Arousal, Valence and their association with Action, Emotion, Perception and Thinking) by a sample of 39 (28 female) participants aged between 18 years and 44 years (*M* = 25.31 years, *SD* = 6.73) in a preceding rating-study (see Table S1 in the Supplementary Materials for descriptive and inferential statistics).

### 2.3. Procedure

#### 2.3.1. Training Sessions

The training sessions took place with one or two participants in one room of the Department of Biological Psychology at Heinrich Heine University Düsseldorf. The training period for each participant included two training sessions on separate days with two training-blocks in each session, run with PsychoPy (version 1.81.03, avaliable online: http://www.psychopy.org/changelog.html#psychopy-1-81-03) [48] on a Fujitsu Lifebook A512. A two-minute break separated the two blocks. In both blocks, all OPic, SPic and NoPic words were presented four times each for 5000 ms in a randomized order. The ISI was set to 500 ms. Within each block, each OPic word was combined once with each of the four pictures of the assigned object taken from different perspectives. Similarly, each SPic word was combined once with each of the four sections of one structure picture. In this way, each OPic and each SPic word appeared four times per block and thus eight times per training and each OPic word could be associated with one object and each SPic word could be associated with one structure. NoPic words were presented as often as the OPic and SPic words, but they appeared alone on the computer screen without any additional picture. Participants were asked to memorize the presented words and, for the OPic and SPic words, their associated pictures. Each block took about 15 min to complete. Participants were told that learning performance checks would be conducted after the training session. At first, free reproduction was assessed followed by a multiple-choice and picture assignment questionnaire (for details see Section 2.3.3).

#### 2.3.2. EEG Session

EEG was acquired individually in an electrically shielded EEG chamber in the department of Biological Psychology at Heinrich Heine University Düsseldorf. During the EEG session, concrete and abstract real words as well as the novel words were presented intermixed in three blocks. The novel words included the 45 words that appeared during the training, as well as the 15 non-trained novel words (New condition). In each block, each novel and real word was presented once, and the order of presentation was randomized.

The participants’ task was to judge whether the real and novel words were either concrete or abstract. This task was chosen because the definitions of concrete and abstract could be applied to both the real and novel words. This made it possible to use the same task for both types of words, which was especially important, as the words appeared intermixed. In the instructions for the participants, concrete was defined as referring to something perceivable via the senses (e.g., sight, touch) including real concrete words as well as the newly learned OPic and SPic words, which referred to the associated picture stimuli. Abstract was defined as referring to something not perceivable via the senses, including real abstract words as well as the NoPic and New words. The latter were introduced as filler stimuli to provide the same number of real and novel words.

Each trial started with a fixation cross with a jittered duration between 1200 ms and 1600 ms. Then the word was presented for 800 ms, followed by a jittered blank screen of 300 ms to 500 ms duration. Afterwards, the assignment of the left and right Ctrl-button of a computer keyboard to the concrete and abstract response option appeared on the screen. The button-response assignment varied randomly between trials to make sure that motor preparation would not confound the recorded ERPs. The inter-trial interval was again randomly jittered between 300 ms and 500 ms. Participants had the possibility to take a self-paced break every 20 trials. Participants were asked to keep their left and right index fingers on the Ctrl-buttons in order to reduce movement artifacts. The EEG task took about 30 min to complete. Subsequent to the EEG task, participants completed the multiple-choice and picture assignment learning performance checks.

#### 2.3.3. Learning Performance Questionnaires

Different questionnaires assessed the participants’ learning performance. A free reproduction task tested the ability to recall the learned words freely after both training sessions. Following each training and the EEG session, a multiple-choice questionnaire with a list of all words tested the participants’ ability to assign the novel words to their category (based on the training condition associated with object, structure and no picture). In an attached picture assignment task, participants were additionally asked to assign each novel word to the printed photographs of the objects/structures. In the learning performance tests, participants could reach one point per correct free reproduction and assignment of the novel words to their category (OPic, SPic, NoPic) or picture (OPic, SPic), respectively. In all versions of the multiple-choice questionnaire, the order of the words was randomized. For each category and learning performance measure, the percentage of correct reproduction and assignments was calculated.

### 2.4. EEG Recording and Preprocessing

#### 2.4.1. Recording

EEG was recorded via 28 silver/silver chloride ring-electrodes, on a textile cap with pre-mounted holders (actiCap; Brainproducts GmbH, Germany) following the extended 10–20 system [49] (electrode sites were F7, F3, Fz, F4, F8, FC5, FC1, FC2, FC6, T7, C3, Cz, C4, T8, CP5, CP1, CP2, CP6, P7, P3, Pz, P4, P8, PO9, O1, Oz, O2, and PO10). Additionally, two electrodes at the outer canthi of the eyes and one above and below the right eye, respectively, recorded horizontal and vertical eye movements. The ground electrode was attached at electrode site AFz and the online reference was attached to the nose. Impedances were kept below 5 kΩ. EEG data were amplified via a BrainAmp DC amplifier (BrainProducts GmbH, Gilching, Germany). The Brain Vision Recorder software (Version 1.20.0506, Brain Products GmbH) was used for data acquisition with a sampling rate of 1000 Hz and an online lowpass filter of 100 Hz on a Windows 10 Dell Intel Premium PC. The software Presentation (Version 17.0, Neurobehavioral Systems Inc., Albany, CA, USA) on a Windows 10 Dell Intel Premium PC controlled the timing of stimulus presentation during the EEG session on a 22′′ LED Dell monitor with 1680 × 1050-pixel resolution and a refresh rate of 60 Hz. The software also recorded the participants’ responses given via a Microsoft USB keyboard.

#### 2.4.2. Preprocessing

EOG electrodes were re-referenced bipolarly and scalp electrodes were referenced to an average reference including all electrodes (C3, C4, CP3, CP4, CPz, Cz, F3, F4, F7, F8, FC3, FC4, FCz, FT7, FT8, Fz, P3, P4, P7, P8, PO3, PO4, PO7, PO8, POz, Pz), except for T7 and T8, which showed extensive muscle artifacts in some participants. Next, data underwent a global direct current detrend [50]. We applied butterworth zero-phase filters with a highpass threshold of 0.5 Hz and a lowpass threshold of 20 Hz, both with 24 dB/Oct. Additionally, a 50 Hz notch-filter was applied. After a classic ICA in semiautomatic mode on 120 s of the data, components including sharp, frontally pronounced positive deflections caused by blinking were detected by visual inspection and removed from the signal via an ICA back transformation. For 18 participants, one single component could be identified depicting the eye blink artifact. For the remaining three participants two or three components were excluded before the back transformation. Continuous data were segmented starting 300 ms pre- and ending 1200 ms post-stimulus onset. After a baseline correction for the 300 ms pre-stimulus interval, an automatic artifact rejection was applied with the following parameters: a maximal allowed voltage step of 50 μV/ms, a maximal/minimal amplitude difference between the highest and the lowest data point of 100/0.1 μV in 100 ms, and a maximally/minimally allowed amplitude of ±100 μV. Subsequently, artifact-free segments were divided into the experimental conditions OPic, SPic, NoPic and New for novel words, and concrete and abstract for real words. In the OPic, SPic and NoPic conditions, only those novel words were included, which participants correctly assigned to their training condition in the multiple-choice questionnaire after the EEG session. This resulted in a mean number of 38.8 trials (*SD* = 7.4) in the OPic, 37.6 trials (*SD* = 7.2) in the SPic and 36.5 trials (*SD* = 8.4) in the NoPic condition entering into the averaged ERP waveforms. In real-word conditions, all artifact-free segments (concrete: *M* = 89.2, *SD* = 2.1; abstract: *M* = 89.0, *SD* = 2.2) entered into the average ERP waveforms.

#### 2.4.3. ERP Analyses

Nine electrodes (F3, Fz, F4, C3, Cz, C4, P3, Pz, P4) equally distributed across the head were chosen for the ERP analyses. ERP time windows were set after visual inspection of the grand average ERP waveforms and in line with previous studies [30,31]. For the N400, the mean amplitude was extracted from the time window between 300 ms and 500 ms. The N700 is more a slow wave rather than a clearly defined ERP component, and the visual inspection of our data suggested different result patterns early and late in the N700 time window between 500 ms and 900 ms. We thus split the time window and analyzed an early N700 (from the 500 ms to 700 ms, compare, e.g., [34,35,51]) and a late N700 (from 700 ms to 900 ms) separately (compare, e.g., [36,52]). Novel words from the New condition were excluded from ERP analyses as they were only introduced as filler stimuli and we were not interested in studying old/new ERP effects, which are typically very pronounced [53,54].

### 2.5. Statistical Data Analyses

Data analysis was conducted with IBM SPSS Statistics (version 23, IBM corporation, Armonk, NY, USA). Behavioral learning and concreteness-judgment performance as well as electrophysiological data were analyzed with different repeated measures analyses of variance (ANOVA). If the Mauchly test indicated a violation of the assumption of sphericity, Greenhouse-Geisser correction was applied and corrected degrees of freedom and *p*-values will be reported. For significance, an α-level of 0.05 was assumed. Post-hoc pairwise comparisons were Bonferroni corrected.

## 3. Results

### 3.1. Behavioral Data

#### 3.1.1. Learning Performance

##### Free Reproduction

**Figure 1:**
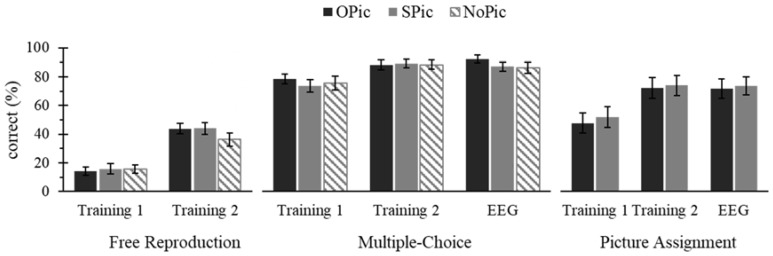
Novel word learning performance. The mean (± one standard error for *n* = 21) learning performance (% correct) for the free reproduction task (**left**); multiple-choice questionnaire (**middle**) and picture assignment (**right**); separately for novel words associated with object (OPic), structure (SPic) or no picture (NoPic). Please note that the latter two learning measures were additionally acquired after the EEG session.

In order to check how well participants learned the novel words, a Category (OPic, SPic, NoPic) × Session (first and second training session) repeated measures ANOVA was conducted on the performance in the free reproduction task (see Figure 1, left). This analysis revealed that the Category did not have a significant effect on the percentage of correct free reproductions, *p* = 0.211. Correct free reproductions significantly increased from the first to the second training session, *F*(1, 20) = 44.607, *p* < 0.001, η_p_² = 0.690. The Category × Session interaction was significant, *F*(1.450, 28.992) = 3.784, *p* = 0.047, η_p_² = 0.159. Paired *t*-tests revealed that the performance increase was significant for all novel word categories, all *p* < 0.001, with the largest increase for OPic, followed by SPic and NoPic.

##### Multiple-Choice

Performance in the multiple-choice test (see Figure 1, middle) served as a second measure of learning of the novel words. This measure was not only applied after each of the two learning sessions, but also after the EEG session. A Category (OPic, SPic, NoPic) × Session (first and second training session, EEG session) repeated measures ANOVA showed that neither the main effect of Category nor the Category × Session interaction were significant for the multiple-choice learning performance, both *p* ≥ 0.078. We found a significant main effect of Session, *F*(1.424, 28.483) = 15.923, *p* < 0.001, η_p_² = 0.443. Pairwise comparisons showed a significant performance increase from the first to the second training session and from the first training to the EEG session, *p* = 0.002 and *p* < 0.001, respectively. The second training session and the EEG session did not differ significantly, *p* = 1.000.

##### Picture Assignment

A Category (OPic, SPic) × Session (first and second training session, EEG session) repeated measures ANOVA was performed on the performance in the picture assignment test (see Figure 1, right), with the aim of determining how well participants learned to associate the novel words with the respective pictures. The ANOVA showed that neither the main effect of Category nor the Category × Session interaction affected the percentage of correct picture-assignments significantly, both *p* ≥ 0.511. Again, the Session had a significant effect, *F*(1.234, 24.673) = 17.753, *p* < 0.001, η_p_² = 0.022. Pairwise comparisons revealed a significant increase in correct assignments from the first to the second training session, and from the first training to the EEG session, *p* = 0.002 and *p* < 0.001, respectively. The second training and EEG session did not differ significantly, *p* = 1.000.

#### 3.1.2. Concreteness-Judgment Task in the EEG Session

Error rates were calculated as the percentage of wrong responses of all given responses and were averaged separately for each experimental condition (real word Concreteness: concrete and abstract; novel word Category: OPic, SPic, NoPic).

##### Error Rates for Real Words

Mean error rates were 2.3% (*SE* = 0.7%) for concrete words and 3.2% (*SE* = 0.7%) for abstract words. A paired *t*-test did not reveal a significant difference between concrete and abstract words, *t*(20) = −1.057, *p* = 0.303.

##### Error Rates for Novel Words

Mean error rates were descriptively smallest in response to OPic (*M* = 8.3%, *SE* = 2.2%), followed by SPic (*M* = 12.3%, *SE* = 2.6%) and NoPic (*M* = 17.3%, *SE* = 4.0%). In a repeated measures ANOVA the effect of the Category on error rates did not reach significance, *p* = 0.068.

### 3.2. Electrophysiological Data

#### 3.2.1. ERP Effects for Real Words

**Figure 2:**
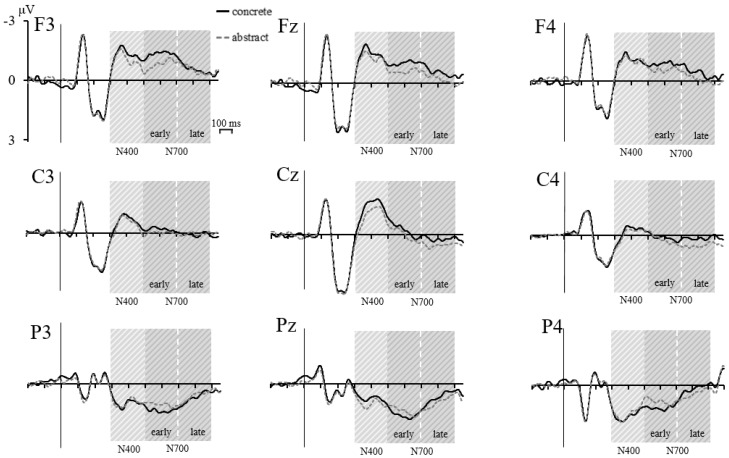
Real word ERPs. Grand averages (*n* = 21) of the ERP waveforms elicited by concrete and abstract words for all analyzed electrodes. The shaded areas mark the time windows of the N400 (300–500 ms), the early N700 (500–700 ms) and late N700 (700–900 ms).

Firstly, we aimed at replicating the well-known CE for real words with our experimental paradigm and setup. Therefore, amplitudes of the N400 and the early and late N700 were analyzed via repeated measures ANOVAs with the factor Concreteness (concrete, abstract) and the topographical factors Frontality (frontal, central, parietal) and Laterality (left, middle, right). Figure 2 shows the grand averages for concrete and abstract words for all analyzed electrodes. Inferential statistics are listed in Table 1. Descriptive statistics (*M*, *SE*) for the amplitudes of the ERP components elicited by concrete and abstract words at each electrode site can be found in Table S2 in the Supplementary Materials. Only main and interaction effects involving the factor Concreteness will be reported in the text.

##### Real Word N400

Concreteness had a significant effect on N400 amplitudes, *p* = 0.006, with higher (i.e., more negative) amplitudes for concrete (*M* = −0.259 μV, *SE* = 0.135 μV) than abstract words (*M* = −0.124 μV, *SE* = 0.128 μV). The Concreteness × Laterality interaction was significant, *p* = 0.023. Subsequent paired *t*-tests comparing abstract and concrete words for each of the three levels of Laterality revealed a significantly higher (i.e., more negative) N400 amplitude for concrete words only at electrodes of the midline (mean difference: −0.274 μV, *SE* = 0.062 μV), *t*(20) = −4.442, *p* < 0.001. The differences were significant at neither the left side, nor at right side electrodes, both *p* ≥ 0.078. Neither the two-way interaction Concreteness × Frontality nor the three-way interaction Concreteness × Frontality × Laterality reached significance, both *p* ≥ 0.060.

##### Real Word N700

Concreteness had a significant effect on early N700 amplitudes, *p* = 0.016, with a higher (i.e., less positive) amplitude for concrete (*M* = 0.068 μV, *SE* = 0.093 μV) than abstract words (*M* = 0.171 μV, *SE* = 0.092 μV). The Concreteness × Frontality interaction was significant, *p* = 0.005. Subsequent paired *t*-tests comparing abstract and concrete words for each of the three levels of Frontality revealed a significantly more negative early N700 amplitude for concrete words at frontal (mean difference: −0.408 μV, *SE* = 0.117 μV), *t*(20) = −3.500, *p* = 0.002, and central electrodes (mean difference: −0.168 μV, *SE* = 0.066 μV), *t*(20) = −2.549, *p* = 0.019. The pattern was inversed at parietal electrodes, where concrete words elicited a significantly more positive amplitude than abstract words (mean difference: 0.270 μV, *SE* = 0.119 μV), *t*(20) = 2.275, *p* = 0.034. Neither the two-way interaction Concreteness × Laterality nor the three-way interaction Concreteness × Frontality × Laterality reached significance, both *p* ≥ 0.363.

Concreteness also had a significant effect on late N700 amplitudes, *p* = 0.002, again with higher (i.e., less positive) amplitudes for concrete (*M* = 0.055 μV, *SE* = 0.064 μV) than abstract words (*M* = 0.252 μV, *SE* = 0.076 μV). The Concreteness × Laterality interaction was significant, *p* = 0.025. Subsequent paired *t*-tests comparing abstract and concrete words for each of the three levels of Laterality revealed a significantly higher late N700 amplitude for concrete words at midline (mean difference: −0.298 μV, *SE* = 0.088 μV), *t*(20) = −3.387, *p* = 0.003, and right side (mean difference: −0.300 μV, *SE* = 0.084 μV), *t*(20) = −3.574, *p* = 0.002 electrodes. Amplitudes elicited by concrete and abstract words did not differ significantly at left side electrodes, *p* = 0.916. Neither the two-way interaction Concreteness × Frontality nor the three-way interaction Concreteness × Frontality × Laterality reached significance, both *p* ≥ 0.656.

#### 3.2.2. ERP Effects for Novel Words

**Figure 3:**
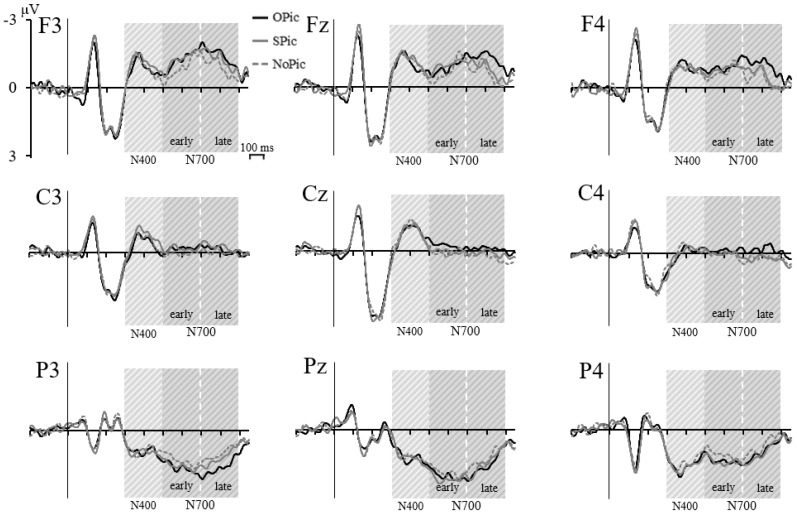
Novel word ERPs. Grand averages (*n* = 21) of the ERP waveforms elicited by novel words associated with object (OPic), structure (SPic) or no pictures (NoPic) for all analyzed electrodes. The shaded areas mark the time windows of the N400 (300–500 ms) and the early N700 (500–700 ms) and late N700 (700–900 ms).

The main analysis examined the effects of the training-induced visual imageability of the novel words on linguistic processing. Repeated measures ANOVAs with the training-induced factor Category (OPic, SPic, NoPic) and the topographical factors Frontality (frontal, central, parietal) and Laterality (left, middle, right) were conducted on the amplitudes of the N400 and the early and late N700. Figure 3 shows the grand averages for OPic, SPic and NoPic words for all analyzed electrodes. Inferential statistics are listed in Table 2. Descriptive statistics (*M*, *SE*) for the ERP components elicited by OPic, SPic and NoPic words at each electrode site can be found in Table S3 in the Supplementary Materials. Only main and interaction effects involving the factor Category will be reported in the text.

##### Novel Word N400

For N400 amplitudes, neither the main effect of Category nor any of its interactions with the topographical factors reached significance, all *p* > 0.399.

##### Novel Word N700

For the early N700, neither the main effect of Category nor any of its interactions with the topographical factors reached significance, all *p* > 0.141.

For the late N700, the two-way interaction Category × Frontality was significant, *p* = 0.014. In order to resolve this interaction, repeated measures ANOVAs investigated the effect of Category separately for each level of Frontality. The Category had a significant effect on the late N700 amplitudes at frontal (*p* = 0.008) and parietal (*p* = 0.043) but not at central (*p* = 0.066) electrode sites. Pairwise comparisons for the frontal electrodes showed that OPic words (*M* = −1.244 μV, *SE* = 0.256 μV) elicited a significantly more negative amplitude than NoPic words (*M* = −0.624 μV, *SE* = 0.227 μV), *p* = 0.021. The comparisons of SPic (*M* = −0.858 μV, *SE* = 0.277 μV) to OPic and NoPic did not reach significance, *p* = 0.087 and *p* = 0.741, respectively. The pattern was inverted at parietal electrodes, where OPic words (*M* = 1.374 μV, *SE* = 0.197 μV) elicited a more positive amplitude than NoPic words (*M* = 0.978 μV, *SE* = 0.172 μV) which was at trend level after Bonferroni correction, *p* = 0.099. Again, the comparisons of SPic (*M* = 1.206 μV, *SE* = 0.214 μV) to OPic and NoPic were not significant, *p* = 0.396 and *p* = 0.571, respectively. The two-way interaction Category × Laterality was also significant, *p* = 0.047. In order to resolve this interaction, repeated measures ANOVAs investigated the effect of Category separately for each level of Laterality. The Category had a significant effect on late N700 amplitudes only at right side electrodes, *p* = 0.027 (left side and midline both *p* ≥ 0.133). Pairwise comparisons for the right-side electrodes showed that OPic words (*M* = −0.075 μV, *SE* = 0.130 μV) elicited a more negative amplitude than SPic words (*M* = −0.235 μV, *SE* = 0.147 μV) and NoPic (*M* = −0.178 μV, *SE* = 0.136 μV) which was at trend level after Bonferroni correction, *p* = 0.076 and *p* = 0.080, respectively, while the latter two did not differ significantly, *p* = 1.000. Neither the Category main effect nor the three-way interaction Category × Frontality × Laterality were significant, both *p* ≥ 0.080.

## 4. Discussion

This study investigated the effect of visual imageability on linguistic processing untainted of lexical concreteness. In a two-day training paradigm, we induced visual imageability by letting participants associate novel words with two qualitatively different kinds of pictures. In a post-training EEG session, which also entailed real concrete and abstract words, we replicated the classical CE for real word processing, with higher (i.e., more negative) N400 and N700 amplitudes for concrete than abstract words. In the early N700 time window (500–700 ms), concrete words elicited significantly more negative amplitudes at frontal and central, but more positive amplitudes at parietal electrode sites. In the late N700 time window (700–900 ms), the CE was modulated by the laterality, with a significant CE at right side and midline, but no significant CE at left side electrodes. Concerning the processing of the novel words, we did not find effects of imageability on N400 amplitudes when comparing novel words associated with pictures and familiarized novel words without any picture association. For the late N700 time window, we found an imageability effect: Novel words associated with pictures of novel objects elicited significantly more negative amplitudes at frontal and more positive amplitudes at parietal electrode sites than non-imageable novel words. The direction of this effect at frontal electrode sites is in line with the hypothesis that a higher imageability contributes to the real word CE at this later conceptual processing stage reflected by the higher N700, while the N400 CE might reflect the interaction of sensorimotor and lexically coded conceptual features.

The higher N400 and N700 amplitudes for concrete in comparison to abstract real words are in line with the well-known CE and underline the suitability of our paradigm to uncover such semantic processing differences. The classical view explains N400 and N700 CEs on the basis of the extended dual-coding theory, namely to reflect a reliance on more easily accessible and qualitatively different information when processing concrete as compared to abstract concepts [10,33]. The concrete words used in the present study were rated higher in concreteness, imageability and their association with action and perception than abstract words (see Table S1 in the Supplementary Materials). The rating scores thus suggest that concrete conceptual representations are based on multi-modal information experienced with the external bodily senses, in line with previous psycholinguistic studies [3]. Hence, stronger semantic integration processes might explain the N400 CE, and stronger mental imagery processes driven by integrated sensory information the N700 CE [35] in our study. We did not find a behavioral CE on error rates, but a dissociation of behavioral and electrophysiological CEs is known from previous literature [36].

In order to interpret the electrophysiological results for the novel word processing against the background of their visual imageability, the validity of the training paradigm has to be examined. The participants’ performance in the assessed learning questionnaires suggests that the training paradigm successfully established an association between the novel words and the assigned pictures. Free reproduction as well as multiple-choice and picture assignment performance showed an increase over the sessions for all three novel word categories. The novel words associated with pictures in our study seem to have additionally profited from their induced imageability, as indicated by the Category × Session interaction effect on the percentage of correct free reproductions. This is in line with another word-learning study, which traced back a learning advantage for concrete over abstract words to a stronger activation in the ventral anterior fusiform gyrus, which is involved in higher order visual processing [55].

The training-induced visual imageability did not affect the N400 amplitudes elicited during the processing of the novel words in the EEG session. As the novel words’ imageability arose from mere visual information, they lacked concreteness in terms of lexically and multi-modally coded information, which seems to be crucial for the N400 CE [35,42]. An alternative explanation might be that the imageability induced for novel words was not sufficiently consolidated to elicit N400 effects. Other word-learning studies did neither find word-like N400 effects after a short training [56] nor before a consolidation period [57], while later ERP effects were found. However, our study consisted of two training sessions on separate days before the EEG acquisition and should thus have provided a sufficiently long period for consolidation. In addition, our analyses were restricted to those words for which the training condition was correctly recognized after the EEG assessment. Furthermore, Palmer, Macgregor and Havelka [29] found an N400 CE for words with merely lexically acquired concreteness (associated to written definitions) at the very same day. Hence, our data can reasonably be interpreted as being consistent with the hypothesis that the N400 CE relies on the interaction of several semantic features, to which isolated visual imageability does not contribute autonomously.

In line with our hypothesis, we found an effect of the training-induced imageability on late N700 amplitudes. This effect interacted with the topographical factors. OPic words elicited a significantly higher late N700 amplitude than NoPic words at frontal electrode sites. Amplitudes elicited by SPic processing were descriptively between those for OPic and NoPic but did not differ significantly from either of them. Only at electrode sites over the right hemisphere, OPic words elicited higher late N700 amplitudes than both SPic and NoPic at trend level. In a recent study on single-word processing, N700 imageability effects only arose when both, the task and the stimuli, afforded them [35]. The N700 result for the real words employed in the present study appears to indicate that the chosen task was appropriate for eliciting imagery processes. Concerning the lateralization of the N700 CE, previous studies yielded inconsistent findings, with more pronounced CEs either over the left [35] or right hemisphere ([34], but at occipital electrode sites), or no laterality difference at all [36,51,52]. However, in our study, the lateralization is in line with the stronger right hemispheric late N700 CE found for real abstract and concrete words.

Our pattern of results for novel words might suggest that the qualitative differences between the two kinds of employed pictures caused the late N700 modulation. The novel object pictures showed unique manmade objects, which formed a distinct entity: this characteristic might underlie advantages in early learning of imageable words [16]. The electron-microscopical pictures, in turn, although also containing coherent elements, were more heterogeneous and clearly less tangible. A possible alternative explanation for the graded late N700 effects might be a systematically weaker association of SPic words with their pictures. This explanation, however, seems implausible regarding the comparable performance in learning of SPic and OPic words. Notably, the deflection in the late N700 time window was positive at parietal electrodes, possibly reflecting a late positive component (LPC), usually interpreted to stand for the recollection of individual experience in linguistic processing [58,59]. In a word-learning study employing an old/new task, LPC amplitudes elicited by novel words were even higher after a consolidation period, while amplitudes decreased for familiar words [60]. The authors suggest that conscious recognition favors novel word learning. In our study, the LPC pattern was also found with more positive early N700 amplitudes elicited by concrete compared to abstract word processing.

As both the frontal N700 and the parietal LPC were delayed for novel (late N700) compared to real word CE (early N700), this might suggest a functional dissociation within the N700 time window. The relatively later N700 modulations by the imageability of novel words in our study might be due to their novelty, which could have led to a delayed processing in comparison to familiar concrete and abstract words. Previous findings concerning the temporal dynamics of the N700 for familiar words, however, are inconsistent, with findings of early onsets around 500 ms (compare e.g., [35,51]) as well as relatively late onsets only after 700 ms (compare e.g., [36]). Thus, exploring the functional role of different stages of the N700 might be a promising approach for further research.

Findings of Barber, et al. [36] challenge the classical interpretation of the N400 and N700 CE. In their ERP study, they controlled for concrete and abstract words’ imageability and other psycholinguistic variables that are known to lead to concrete word processing advantages (i.e., familiarity, age of acquisition and context availability) and still found higher N400 and N700 amplitudes for concrete words. They suggest that the CE in these two ERP components is rather modulated by the degree of multimodality inherent to the underlying conceptual information. Following this suggestion, the lacking effect of the novel word category on N400 amplitudes might be explained by the unimodal visual information the words received during the training. At a later stage of semantic processing, additional information arising from mental imagery might have been processed, leading to effects of the novel word category in the late N700 time window. The tangible appearance of the novel object pictures might have led to an impression of an affordance inherent to graspable objects [61,62] despite the lack of former experience with them. Linguistic processing might rely on this information [62,63], but rather at a later, more explicit processing stage, reflected by the N700.

In former studies, the electrophysiological CE could not easily be attributed to either the words’ concreteness or imageability, as the two variables are highly correlated [3], and in most studies the terms were either used interchangeably or alone without controlling the other (for a counterexample see [36]). By modulating the visual imageability of former meaningless, novel words in a word learning paradigm, and thereby ruling out any possible confounds of word concreteness and other psycholinguistic variables, this study delivers insights into the isolated effect of words’ imageability on linguistic processing. It seems that mere visual imageability plays a role at later explicit imagery processing stages (N700) but not in automatic semantic feature integration (N400). The effects in the N700 time window might also be explained by additional multi-modal information introduced by the novel object pictures and processed during mental imagery, which are not available at automatic stages of semantic processing.

## Figures and Tables

**Table 1:** Real Word Analyses. Full inferential statistics of the 2 × 3 × 3 repeated measures ANOVAs on N400, early N700 and late N700 amplitudes.

Effect	*df*	*F*	*p*	η_p_²
N400 (300–500 ms)				
Concreteness	1, 20	9.566	0.006	0.324
Concreteness × Frontality	1.251, 25.023	0.472	0.540	0.023
Concreteness × Laterality	1.302, 26.036	5.203	0.023	0.206
Concreteness × Frontality × Laterality	4, 80	2.363	0.060	0.106
Frontality	1.135, 22.691	21.042	<0.001	0.513
Laterality	2, 40	5.614	0.007	0.219
Frontality × Laterality	2.544, 50.884	2.136	0.116	0.096
early N700 (500–700 ms)				
Concreteness	1, 20	6.961	0.016	0.258
Concreteness × Frontality	1.173, 23.451	8.588	0.005	0.300
Concreteness × Laterality	2, 40	1.039	0.363	0.049
Concreteness × Frontality × Laterality	4, 80	1.019	0.403	0.048
Frontality	1.364, 27.271	37.281	<0.001	0.651
Laterality	2, 40	0.849	0.435	0.041
Frontality × Laterality	1.643, 32.859	3.326	0.057	0.143
late N700 (700–900 ms)				
Concreteness	1, 20	12.796	0.002	0.390
Concreteness × Frontality	1.142, 22.845	0.247	0.656	0.012
Concreteness × Laterality	1.346, 26.920	4.974	0.025	0.199
Concreteness × Frontality × Laterality	4, 80	0.187	0.944	0.009
Frontality	1.314, 26.286	21.153	<0.001	0.514
Laterality	2, 40	1.456	0.245	0.068
Frontality × Laterality	2.178, 43.553	7.345	0.001	0.269

**Table 2:** Novel word analyses. Full inferential statistics of the 3 × 3 × 3 repeated measures ANOVA on N400, early N700 and late N700 amplitudes as well as repeated measures ANOVAs with the factor Category (OPic, SPic, NoPic) resolving the significant interactions.

Effect	*df*	*F*	*p*	η_p_²
N400 (300–500 ms)				
Category	2, 40	0.559	0.576	0.027
Category × Frontality	2.242, 44.834	0.135	0.895	0.007
Category × Laterality	4, 80	1.026	0.399	0.049
Category × Frontality × Laterality	8, 160	0.858	0.554	0.041
Frontality	1.138, 22.768	21.611	<0.001	0.519
Laterality	2, 40	2.387	0.105	0.107
Frontality × Laterality	2.351, 47.021	1.924	0.151	0.088
early N700 (500–700 ms)				
Category	2, 40	0.556	0.578	0.027
Category × Frontality	2.115, 42.309	2.033	0.141	0.092
Category × Laterality	4, 80	0.415	0.797	0.020
Category × Frontality × Laterality	5.126, 102.523	1.214	0.308	0.057
Frontality	1.198, 23.956	28.031	<0.001	0.584
Laterality	1.328, 26.560	1.150	0.311	0.054
Frontality × Laterality	1.747, 34.947	4.832	0.017	0.195
late N700 (700–900 ms)				
Category	2, 40	2.698	0.080	0.119
Category × Frontality	1.912, 38.243	4.917	0.014	0.197
Category ^a^: Repeated measures ANOVA				
frontal	2, 40	5.450	0.008	0.214
central	1.567, 31.341	3.185	0.066	0.137
parietal	2, 40	3.408	0.043	0.146
Category × Laterality	4, 80	2.533	0.047	0.112
Category ^a^: Repeated measures ANOVA				
left side	2, 40	1.189	0.315	0.056
midline	1.509, 30.187	2.255	0.133	0.101
right side	2, 40	3.941	0.027	0.165
Category × Frontality × Laterality	4.367, 87.338	0.395	0.828	0.019
Frontality	1.161, 23.225	27.382	<0.001	0.578
Laterality	1.500, 30.009	0.807	0.423	0.039
Frontality × Laterality	2.164, 43.278	12.682	<0.001	0.388

^a^ Resolutions of the significant interactions by repeated measures ANOVA with the factor Category.

## Supplementary Materials

Supplementary materials can be found at http://www.mdpi.com/2227-9059/6/3/75/s1.
