# Supplementary material for: The Effect of Training-Induced Visual Imageability on Electrophysiological Correlates of Novel Word Processing
Source: Biomedicines. 2018 Jul 1;6(3):75. doi: 10.3390/biomedicines6030075 (PMC6165368; doi:10.3390/biomedicines6030075)
Supplement: Supplementary file 1 [file biomedicines-06-00075-s001.zip › Supplementary_TableS1.pdf]

The effect of training-induced visual imageability on electrophysiological correlates of novel word processing

Supplementary material

Table S1

Psycholinguistic variables of the real concrete and abstract words rated in a pre-study.

Scale	Concreteness	*M*	*(SE)*	*df*	*t*	*p*
Concreteness	concrete	6.356	(0.028)	46.024	57.018	< .001
	abstract	3.096	(0.05)			
Imageability	concrete	6.662	(0.033)	39.787	44.067	< .001
	abstract	3.062	(0.075)			
Arousal	concrete	2.937	(0.142)	50.746	-3.724	< .001
	abstract	3.888	(0.212)			
Valence	concrete	0.224	(0.082)	34.614	-0.597	.555
	abstract	0.389	(0.264)			
Action	concrete	4.498	(0.196)	50.73	7.106	< .001
	abstract	2.822	(0.131)			
Emotion	concrete	2.176	(0.057)	34.85	-10.911	< .001
	abstract	4.219	(0.178)			
Perception	concrete	3.463	(0.087)	58	2.127	.038
	abstract	3.162	(0.112)			
Thinking	concrete	1.737	(0.079)	47.014	-17.017	< .001
	abstract	4.394	(0.134)			

Note. Mean (*M*) ratings ± one standard error (*SE*) of the ratings on eight different scales (from 1 to 7, except for valence from -3 to +3) for the 30 concrete and 30 abstract words provided by 39 separate raters. Degrees of freedom (*df*), statistic of the independent t-test (*t*) and significance values (*p*) are provided on the right. For Action, Emotion, Perception and Thinking, participants rated the strength of the words' association with these aspects.
