# Supplementary material for: The Effect of Training-Induced Visual Imageability on Electrophysiological Correlates of Novel Word Processing
Source: Biomedicines. 2018 Jul 1;6(3):75. doi: 10.3390/biomedicines6030075 (PMC6165368; doi:10.3390/biomedicines6030075)
Supplement: Supplementary file 1 [file biomedicines-06-00075-s001.zip › Supplementary_TableS2.pdf]

The effect of training-induced visual imageability on electrophysiological correlates of novel word processing

Table S2

Descriptive statistics of ERP amplitudes elicited by real concrete and abstract words.

Frontality	Concreteness	Laterality left	midline	right
N400 (300 - 500 ms)				
Frontal	concrete	-1.254 (0.319)	-1.356 (0.322)	-0.990 (0.324)
	abstract	-0.992 (0.288)	-1.170 (0.297)	-0.951 (0.285)
Central	concrete	-0.511 (0.185)	-1.262 (0.295)	-0.175 (0.256)
	abstract	-0.396 (0.196)	-0.873 (0.264)	-0.086 (0.226)
Parietal	concrete	0.978 (0.273)	0.729 (0.347)	1.508 (0.322)
	abstract	0.969 (0.259)	0.975 (0.306)	1.406 (0.296)
early N700 (500 - 700 ms)				
Frontal	concrete	-1.320 (0.287)	-0.990 (0.226)	-0.843 (0.197)
	abstract	-0.794 (0.262)	-0.571 (0.241)	-0.564 (0.232)
Central	concrete	-0.172 (0.144)	-0.218 (0.237)	0.118 (0.175)
	abstract	0.003 (0.186)	0.010 (0.217)	0.220 (0.163)
Parietal	concrete	1.349 (0.206)	1.556 (0.215)	1.136 (0.160)
	abstract	1.046 (0.221)	1.369 (0.163)	0.817 (0.179)
late N700 (700 - 900 ms)				
Frontal	concrete	-0.606 (0.166)	-0.561 (0.191)	-0.419 (0.168)
	abstract	-0.556 (0.193)	-0.231 (0.189)	-0.111 (0.188)
Central	concrete	0.071 (0.114)	0.209 (0.166)	0.129 (0.120)
	abstract	0.061 (0.151)	0.535 (0.170)	0.503 (0.134)
Parietal	concrete	0.704 (0.136)	0.712 (0.156)	0.259 (0.083)
	abstract	0.638 (0.176)	0.951 (0.170)	0.477 (0.141)

Note. Mean amplitudes in μV (± one standard error) of the N400, early N700 and late N700 elicited by the real concrete and abstract words.
