# Supplementary material for: The Effect of Training-Induced Visual Imageability on Electrophysiological Correlates of Novel Word Processing
Source: Biomedicines. 2018 Jul 1;6(3):75. doi: 10.3390/biomedicines6030075 (PMC6165368; doi:10.3390/biomedicines6030075)
Supplement: Supplementary file 1 [file biomedicines-06-00075-s001.zip › Supplementary_TableS3.pdf]

The effect of training-induced visual imageability on electrophysiological correlates of novel word processing

Table S3

Descriptive statistics of ERP amplitudes elicited by novel OPic, SPic and NoPic words.

Frontality	Category	Laterality left	midline	right
N400 (300 - 500 ms)				
Frontal	OPic	-0.823 (0.342)	-0.980 (0.346)	-0.815 (0.322)
	SPic	-1.024 (0.369)	-1.068 (0.335)	-0.729 (0.318)
	NoPic	-0.817 (0.260)	-1.052 (0.276)	-0.762 (0.314)
Central	OPic	-0.286 (0.178)	-0.699 (0.320)	0.021 (0.278)
	SPic	-0.581 (0.240)	-0.663 (0.255)	-0.001 (0.239)
	NoPic	-0.379 (0.176)	-0.793 (0.286)	-0.079 (0.280)
Parietal	OPic	0.945 (0.321)	1.190 (0.362)	1.525 (0.316)
	SPic	0.966 (0.286)	1.186 (0.252)	1.557 (0.315)
	NoPic	0.889 (0.298)	1.124 (0.332)	1.384 (0.255)
early N700 (500 - 700 ms)				
Frontal	OPic	-1.247 (0.339)	-0.852 (0.348)	-0.902 (0.281)
	SPic	-1.264 (0.364)	-0.944 (0.328)	-0.813 (0.342)
	NoPic	-0.885 (0.313)	-0.729 (0.316)	-0.707 (0.330)
Central	OPic	-0.160 (0.212)	-0.210 (0.275)	-0.134 (0.225)
	SPic	-0.144 (0.224)	0.050 (0.211)	0.012 (0.243)
	NoPic	0.064 (0.230)	0.089 (0.241)	0.037 (0.241)
Parietal	OPic	1.672 (0.295)	2.027 (0.285)	1.366 (0.247)
	SPic	1.574 (0.293)	2.127 (0.266)	1.372 (0.274)
	NoPic	1.326 (0.302)	1.886 (0.231)	1.122 (0.222)
late N700 (700 - 900 ms)				
Frontal	OPic	-1.416 (0.305)	-1.293 (0.284)	-1.022 (0.245)
	SPic	-1.241 (0.309)	-0.809 (0.315)	-0.525 (0.305)
	NoPic	-0.895 (0.294)	-0.619 (0.272)	-0.360 (0.235)
Central	OPic	-0.109 (0.160)	-0.092 (0.223)	-0.170 (0.182)
	SPic	-0.240 (0.203)	0.119 (0.257)	0.219 (0.203)
	NoPic	-0.002 (0.187)	0.277 (0.244)	0.202 (0.195)
Parietal	OPic	1.626 (0.221)	1.530 (0.270)	0.967 (0.174)
	SPic	1.195 (0.195)	1.412 (0.308)	1.009 (0.219)
	NoPic	0.992 (0.181)	1.252 (0.244)	0.691 (0.191)

Note. Mean amplitudes in μV (± one standard error) of the N400, N700a and N700b elicited by novel OPic, SPic and NoPic words.
